# sCD40L联合长春瑞滨对肺腺癌A549细胞的作用

**DOI:** 10.3779/j.issn.1009-3419.2010.07.06

**Published:** 2010-07-20

**Authors:** 珊 刘, 波 陈, 燕 李, 卫红 赵, 剑卿 吴

**Affiliations:** 210029 南京，南京医科大学第一附属医院老年呼吸科 Department of Geriatrics, the First Afﬁliated Hospital of Nanjing Medical University, Nanjing 210029, China

**Keywords:** 可溶性CD40配体, 长春瑞滨, 肺肿瘤, sCD40L, Vinorelbine, Lung neoplasms

## Abstract

**背景与目的:**

CD40信号对肿瘤细胞化疗敏感性的影响存在争议。本研究旨在观察可溶性CD40配体（sCD40L）联合长春瑞滨对肺腺癌A549细胞的作用。

**方法:**

以sCD40L处理A549细胞，MTT法和流式细胞仪检测CD40激发前后长春瑞滨作用下A549细胞的生长、细胞周期及凋亡率等生物学行为的变化，同时用Caspase-3检测试剂盒分析Caspase-3活性的改变。

**结果:**

sCD40L对CD40分子的激发可增强长春瑞滨对表达CD40肺癌细胞株A549增殖的抑制率（*P* < 0.05），但并不显著影响细胞周期时相和细胞凋亡率（*P* > 0.05），Caspase-3活性反而显著下降（*P* < 0.05）。

**结论:**

以sCD40L激发CD40信号可增强肺腺癌细胞A549对长春瑞滨的敏感性，可能通过非凋亡的、Cas-pase非依赖性的机制，且可能与抑制细胞周期进程无关。

## Abstract (en)

**Background and objective:**

The results of published data on the effect of CD40 signal related to cancer cell sensitivity to chemotherapy are inconclusive. The aim of this study is to investigate the effect of soluble CD40 ligand (sCD40L) combined with vinorelbine on lung adenocarcinoma cell line A549.

**Methods:**

Lung adenocarcinoma A549 cells were chosen as target cells and CD40 signal was stimulated by sCD40L. MTT assay and flow cytometry were used to determine the changes of cell proliferation, cell cycle and apoptosis of A549 cells treated by vinorelbine after CD40 was stimulated. The activity of Caspase-3 was measured using Caspase-3 cellular activity assay kit plus.

**Results:**

After CD40 stimulation, an increase of inhibition on CD40 positive cell line A549 proliferation was confirmed when vinorelbine was added (*P* < 0.05). However, no significant changes were shown in apoptosis and cell cycle (*P* > 0.05). On the other hand, the activity of Caspase-3 was substantially decreased (*P* < 0.05).

**Conclusion:**

Stimulation of CD40 can increase lung adenocarcinoma cell line A549 sensitivity to vinorelbine, which may be through a non-apoptosis, Caspase independent mechanism, and not associated with the inhibition of cell cycle.

CD40分子属于肿瘤坏死因子受体（tumor necrosis family receptor, TNFR）超家族成员。CD40及其配体CD40L（CD154）形成的共刺激信号在体内免疫激活过程中起着核心作用。除了免疫细胞外，CD40还表达于多种肿瘤细胞。研究证实，CD40信号的活化在体外对多种肿瘤细胞均有生长抑制作用。有关激发型CD40单抗的临床试验已初步显示一定的作用。CD40相关的治疗已成为肿瘤免疫治疗和基因治疗的发展方向之一^[1-4]^。

近年来研究^[5-9]^发现，CD40信号可影响肿瘤细胞对化疗药物的敏感性，且在不同的肿瘤类型﹑细胞系及药物间表现出一定的差异，但具体机制不明。关于CD40信号对肺癌细胞化疗敏感性的影响鲜有报道，本研究旨在观察可溶性CD40配体（sCD40L）联合长春瑞滨（vinorelbine）对肺腺癌A549细胞的作用，并探讨其可能的机制。

## 1 材料与方法

### 1.1 材料

肺腺癌细胞株A549购自中国科学院上海细胞研究所。RPMI-1640培养基为Gibco公司产品，胎牛血清（FCS）为杭州四季青公司产品，PE标记鼠抗人CD40单克隆抗体﹑鼠抗人IgG1同型对照抗体及sCD40L均为Peprotech公司产品，四氮唑盐（MTT）购自美国Sigma公司，Caspase-3活性检测试剂盒购自南京凯基公司。长春瑞滨为江苏豪森药业有限公司生产。EP ICS XL型流式细胞仪为美国Beckman Coulter公司产品，550酶标仪为美国Biorad公司产品，生物倒置显微镜为Olympus CKX4。

### 1.2 方法

#### 1.2.1 细胞培养

细胞置于含10% FCS的RPMI-1640培养基中，37 ℃、5%CO_2_温箱培养，4 d-5 d传代1次。每次实验前取状态良好的对数生长期细胞，经0.25%胰蛋白酶消化后备用。

#### 1.2.2 细胞表面CD40表达的检测

取A549细胞3×10^5^个细胞与CD40单抗及相应的同型对照小鼠IgG在4 ℃孵育30 min。用含0.25% FCS和0.01% NaN_3_的PBS缓冲液洗涤2次后，重悬细胞并经流式细胞仪检测细胞表面CD40的表达。

#### 1.2.3 MTT比色法检测细胞增殖

取对数生长期的A549细胞（1×10^4^个/孔）加入96孔培养板中，加入CD40L（2 μg/mL），用含10% FCS的RPMI-1640培养基调整培养体积为100 μL，置温箱中培养72 h后更换培养基并加入长春瑞滨（20 μg/mL），每组设8个复孔，同时设空白调零组、细胞对照组，继续培养24 h。在终止培养前4 h每孔加入MTT（5 mg/mL）20 μL，培养终止后，弃去上清液，盐酸异丙醇（每孔100 μL）彻底溶解甲瓒颗粒，酶标仪570 nm处读取吸光度（*A*）值。细胞生长抑制率（%）=（1实验组*A*值/对照组平均*A*值）×100%。

#### 1.2.4 流式细胞术检测细胞周期及凋亡率

取对数生长期的A549细胞（5×10^4^个/孔)加入24孔培养板中，加CD40L（0.2 μg/mL, 1.0 μg/mL, 2.0 μg/mL），用含10%FCS的RPMI-1640培养基调整培养体积为1 mL，置温箱中培养72 h后更换培养基并加入长春瑞滨（20 μg/mL），继续培养24 h，以0.25%胰酶消化并收集细胞，共同预冷的PBS洗2次，加入75%乙醇，-20 ℃固定过夜。细胞离心后PBS洗1次，加入RNA酶（1 mg/mL）、PI（10 μg/mL）溶液、0.5% TritonX-100，室温避光温育30 min，加PBS，EPICS XL流式细胞仪检测分析。实验重复3次。

#### 1.2.5 Caspase-3活性的检测

取对数生长期人肺腺癌细胞A549，制成1×10^6^个/L的细胞悬液，接种于6孔板，加CD40L（2.0 μg/mL），用含10% FCS的RPMI-1640培养基调整培养体积为1 mL，置温箱中培养72 h后更换培养基并加入长春瑞滨（20 μg/mL），同时设细胞对照组﹑ sCD40L和长春瑞滨单独作用组，继续培养24 h，用PBS洗涤细胞2次（离心2 000 rpm，5 min）收集3×10^6^个-5×10^6^个细胞，按照Caspase-3检测试剂盒说明书进一步操作，酶标仪λ=405 nm测定其吸光值。通过计算OD诱导剂/OD阴性对照的倍数来确定凋亡诱导剂组Caspase-3活化程度。

### 1.3 统计学分析

数据分析采用Stata 7.0软件，数据以Mean± SD表示，组间比较采用*t*检验和方差分析，以*P* < 0.05为差异具有统计学意义。

## 2 结果

### 2.1 sCD40L联合长春瑞滨对细胞生长的影响

A549细胞表面CD40分子的表达率为（48.1±6.2）%。显微镜下见sCD40L联合长春瑞滨有最佳的抗A549细胞作用（图 1）。MTT比色法结果显示，sCD40L可抑制A549细胞的体外增殖（*P* < 0.05）；经sCD40L预处理后，长春瑞滨对A549细胞的生长抑制率上调（*P* < 0.05）（表 1）。

**1:**
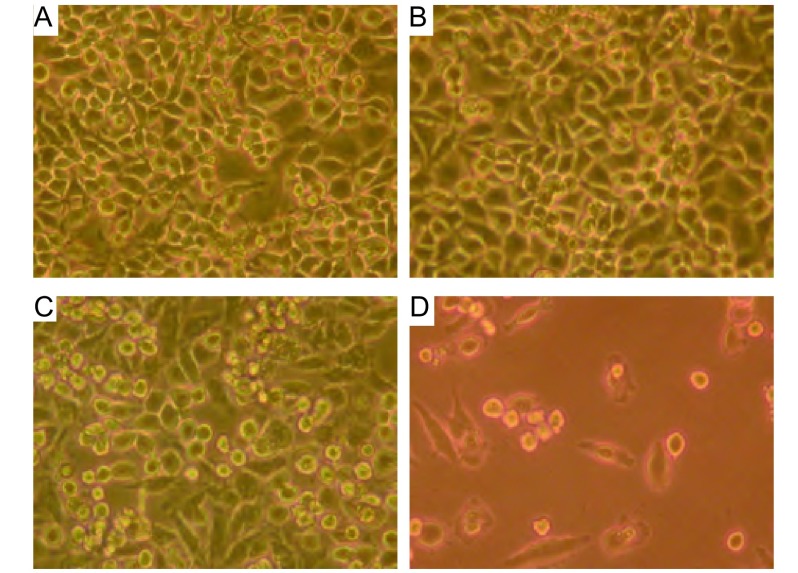
CD40激发前后长春瑞滨对A549细胞作用的镜下观察。A﹑B﹑C﹑D：分别为各组中所观察的一个有代表性的视野。长春瑞滨可导致细胞变小、变圆、脱壁、存活细胞明显减少，其中以sCD40L联合长春瑞滨组最显著。A：空白对照组；B：sCD40L组；C：长春瑞滨组；D：sCD40L联合长春瑞滨组。 Effect of sCD40L combined with vinorelbine on the proliferation of A549 cells observed by microscope. A, B, C or D showed a representative microscope field. The number of survival cells was obviously reduced when vinorelbine was added accompanied shrinking of the cell volume, emerging of a spherical shape and falling off of cells from the culture capsule wall, and this phenomena was most obvious in the group of sCD40L combined with vinorelbine. A: blank; B: sCD40L; C: vinorelbine; D: sCD40L+vinorelbine.

**1:** CD40激发前后长春瑞滨对A549细胞增殖的抑制作用(*n*=3, Mean±SD) Effect of sCD40L combined with vinorelbine on the proliferation of A549 cells (*n*=3, Mean±SD)

Group	Control		Experimental	
	Absorbance (*A*)		Absorbance (*A*)	Inhibition rate (%)
Undisposed	3.282±0.353		2.726±0.349	16.94±10.63
sCD40L	3.131±0.341^*^		2.579±0.356^*^	22.18±9.45^*^
Pretreated with sCD40L *vs* undisposed, ^*^P < 0.05.				

### 2.2 sCD40L联合长春瑞滨对细胞周期及凋亡的影响

相同长春瑞滨剂量下，s CD40L预处理与否对A549细胞周期时相影响不大，周期各时相的百分率无明显变化（*P* > 0.05）；sCD40L预处理后并不显著增加长春瑞滨诱导的细胞凋亡率（*P* > 0.05）。在各组间，细胞周期各时相的百分率及凋亡率的差异无统计学意义（*P* > 0.05）（图 2，图 3）。

**2:**
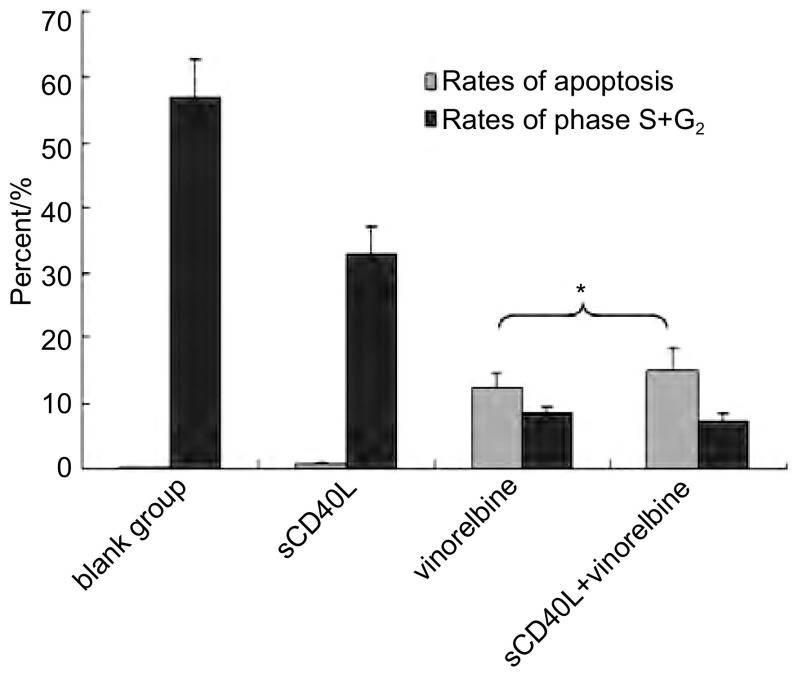
CD 40激发前后长春瑞滨对A549凋亡率及细胞周期的影响（^*^3个sCD40L浓度所得结果的Mean±SD，与长春瑞滨单药组比较，*P* > 0.05） Effect of sCD40L combined with vinorelbine on the proliferation of A549 cells Fig 2 Effect of sCD40L combined with vinorelbine on the cell cycle and apoptosis of A549 cells (^*^Mean values of the results of three sCD40L concentration groups, sCD40L+vinorelbine *vs* vinorelbine alone, *P* > 0.05).

**3:**
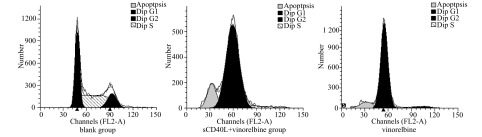
PI染色细胞周期及凋亡情况测定 Cells were analyzed for different phases and apoptotic rates by PI staining

### 2.3 sCD40L联合长春瑞滨对Caspase-3活性的影响

sCD40L单独作用对A549细胞的Caspase-3活性无明显影响；相同的长春瑞滨剂量下，经sCD40L预处理后的A549较未激发组细胞的Caspase-3活性显著下降（*P* < 0.05）（图 4）。

**4:**
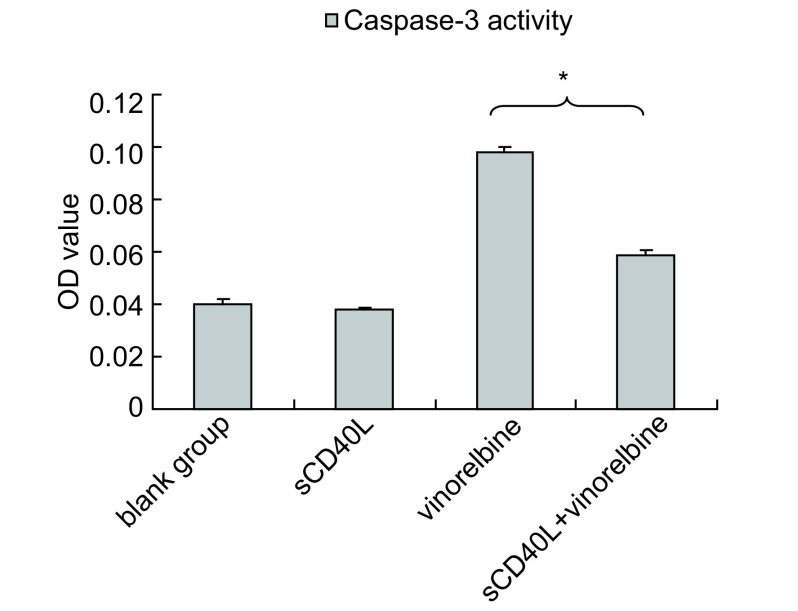
CD40激发前后长春瑞滨对A549细胞中Caspase-3活性的影响 Effect of sCD40L combined with vinorelbine on the Caspase-3 activity of A549 cells (%, *n*=3, Mean±SD) sCD40L+vinorelbine *vs* vinorelbine alone, ^*^P < 0.05.

## 3 讨论

目前，关于CD40信号通路在恶性肿瘤中的生物学作用存在不同意见。一方面，尽管大量研究证实激活CD40信号可抑制多种血液系统肿瘤细胞及乳腺癌﹑大肠癌和肺癌等实体肿瘤细胞的增殖，但也有CD40通路是肿瘤生长信号的报道^[1]^。另一方面，一些研究发现激活CD40信号可增强化疗药物对肿瘤细胞的杀伤力，而另一些研究报道CD40通路与肿瘤细胞耐药有关^[5-9]^。这些矛盾的结论可能源于以下原因：不同肿瘤类型间固有的差异；同种肿瘤中来源不同的细胞株；药物作用机制不同产生的差异；未知的影响因素；激活CD40信号所使用方法的不同，这也是最显著的原因，例如研究发现sCD40L和激发性CD40单抗（agonist）单独作用并不显著增加肿瘤细胞凋亡，除非细胞的蛋白质合成被某些化疗药物所抑制；而由细胞表达的膜型CD40L有明显的促凋亡作用，但却可能抑制化疗药物引起的凋亡^[10-13]^。鉴于CD40信号相关生物学作用的复杂性，有必要针对肺癌细胞进一步深入研究。

本研究中，我们发现sCD40L对CD40的激活可以显著增强长春瑞滨对A549细胞的作用，进一步研究提示其作用机制与促凋亡无关。但有关其它肿瘤细胞的研究中，CD40信号与凋亡的关系被认为是其影响药物敏感性的核心机制，激发CD40信号可能促进凋亡也可能抑制凋亡^[5, 7, 8]^。王天立等^[9]^发现激发CD40信号可使顺铂（DDP）或丝裂霉素（MMC）诱导的非凋亡性细胞死亡显著增加。李旭鹿等^[14]^发现大豆异黄酮（soybean isoflavone, SIF）对长春瑞滨有增效作用，但也与诱导凋亡无关，细胞为变性样改变。付校等^[15]^发现自噬在长春瑞滨诱导的A549细胞死亡中发挥着重要作用。此外，本研究还发现sCD40L联合长春瑞滨较单用长春瑞滨对A549细胞周期时相影响不大，排除了CD40信号通过抑制细胞周期而达到增效的可能。因此，我们推测sCD40L的药物增敏作用可能与其它细胞死亡或细胞生长抑制机制有关。

长期以来，诱导凋亡被认为是化疗药物杀灭肿瘤细胞的主要机制，但越来越多的证据^[16-20]^显示，其它如自噬（autophagy）﹑凋亡样或坏死样程序性死亡（apoptosis-like and necrosis-like programmed cell death）﹑有丝分裂灾难（mitotic catastrophe）以及加速的细胞老化（senescence）等广泛参与了化疗药物的治疗作用。Caspase家族在介导细胞凋亡的过程中起着非常重要的作用，其中Caspase-3为关键的执行分子，它在凋亡信号传导的许多途径中发挥功能，其活性在凋亡发生时常明显升高^[21]^。本研究发现，尽管sCD40L并不明显影响药物诱导的细胞凋亡，但Caspase-3的活性却意外地显著下降。Voorzanger-Rousselot等^[7]^在乳腺癌细胞系中的研究有类似发现：CD40信号的激活可显著抑制化疗药物诱导的细胞凋亡，但Caspase-3/7的活性反而增加2倍-4倍，表明Caspase的活性与细胞凋亡不存在固定的因果关系，可能有其它调节机制。此外，Caspase-3的活性在胞膜不完整的死亡细胞中通常是明显下降的^[21]^，这也进一步提示本研究中sCD40L的增敏作用可能以诱导非凋亡性细胞死亡为主。这些死亡机制均是Caspase非依赖性的，可能与Cathepsins及Calpains等蛋白酶有关，并且广泛存在于以微管为靶点的药物作用中^[22, 23]^。

综上，本研究提示sCD40L对CD40信号的活化可能通过凋亡及细胞周期抑制以外的机制影响长春瑞滨对A549细胞的作用。未来的研究应进一步验证这些潜在机制，并找出可行的调节方法。
